# Lenalidomide Treatment for Multiple Myeloma: Systematic Review and Meta-Analysis of Randomized Controlled Trials

**DOI:** 10.1371/journal.pone.0064354

**Published:** 2013-05-14

**Authors:** Bo Yang, Rui-li Yu, Xiao-hua Chi, Xue-chun Lu

**Affiliations:** 1 Department of Geriatric Hematology, Chinese PLA General Hospital, Beijing, China; 2 Institute of Otorhinolaryngology, Chinese PLA General Hospital, Beijing, China; 3 Department of Pharmacy, the Second Artillery General Hospital, Beijing, China; Istituto di Ricerche Farmacologiche Mario Negri, Italy

## Abstract

**Background:**

In recent years, a number of randomized controlled trials (RCTs) have reported on lenalidomide as a treatment for multiple myeloma (MM). Herein, we report results of a meta-analysis of RCTs examining the efficacy and safety of lenalidomide for MM.

**Patients and Methods:**

Databases were searched using the terms “lenalidomide or revlimid AND multiple myeloma.”RCTs evaluating initial or maintenance therapeutic outcomes were included. Main outcome measures were response rates, progression-free survival (PFS), overall survival, and adverse events.

**Results:**

Seven trials were included (N = 192–614 participants). Lenalidomide doses and treatment regimens differed between trials. Complete response (CR) and very good partial response (VGPR) risk ratios (RR) favored lenalidomide over placebo (CR = 2.54, 95% confidence interval [CI] = 1.29–5.02; VGPR = 2.82, 95% CI = 1.30–6.09). The PFS hazard ratio favored lenalidomide over placebo (0.37, 95% CI = 0.33–0.41). For adverse events, neutropenia, deep vein thrombosis (DVT), infection, and hematologic cancer RR favored placebo over lenalidomide (neutropenia: 4.74, 95% CI = 2.96–7.57; DVT: 2.52; 95% CI: 1.60–3.98; infection: 1.98; 95% CI: 1.50–2.62; hematologic cancer: 3.20; 95% CI: 1.28–7.98).

**Conclusions:**

Lenalidomide is an effective treatment for MM; however, treatment-related adverse events must be considered and appropriate adjustments and/or prophylactic treatment should be initiated where possible.

## Introduction

Multiple myeloma (MM) is a hematological cancer characterized by the malignant proliferation of monoclonal plasma cells in the bone marrow [1], [2]. The worldwide incidence of MM (age-standardized) has been estimated to be 1.7 men and 1.2 women per 100,000 individuals per year [3], most prevalent among older adults between the ages of 65 and 70 years [2]. Mortality worldwide is estimated to be 1.1 men and 0.9 women per 100,000 individuals worldwide [3]. Unfortunately, there is currently no cure for MM. Hence, the aim of treatment for MM is to induce and maintain remission for as long as possible, thereby increasing the length of survival.

Care of patients with MM is complex and focuses on treating the disease process and associated complications [4]. A number of therapeutic approaches and treatment combinations have been employed in the treatment of MM, relying primarily on high dose chemotherapy and autologous stem-cell transplantation [5], maintenance therapy using drug regimens such as alternate-day prednisone [6], and high-dose chemoradiotherapy [7]. However, with these approaches, the response rates and survival times did not differ between patients designated as either high- or low-risk according to M protein values and the symptoms or presence of bone disease; and early treatment did not benefit asymptomatic subjects nor did delayed treatment improve treatment efficacy and survival [8]. The increased ability to precisely identify prognostic factors such as cytogenic abnormalities and to determine risk has increased the individualization of treatment for MM, improving patient response and survival [8]. The incorporation of immunomodulators such as thalidomide, and proteasome inhibitors such as bortezomib into treatment regimens has improved the survival of patients with MM [9], [10]. Treatment with thalidomide, however, is often associated with toxicity that limits its long-term use [11], [12]. Single-agent clinical activity of these newer drugs has been limited and most patients still relapse [13], so the search continues for more effective combinations of drugs or drugs with new mechanisms of action. In 2011, the multiple myeloma guidelines of the National Comprehensive Cancer Network (NCCN) introduced several combinations of drugs for primary induction therapy: 1) the combination of bortezomib/cyclophosphamide/dexamethasone for transplant candidates; 2) the combination of bortezomib/dexamethasone for patients who are not candidates for transplantation; and the combination of melphalan/prednisone/lenalidomide for nontransplant candidates [14].

Lenalidomide, an analogue of thalidomide, appears to be equally efficacious and less toxic than thalidomide [11]. Lenalidomide differs from thalidomide by a single carbonyl ring and an amino acid group [15]. Mechanistically, lenalidomide inhibits proliferation of tumor cells and induces apoptosis, as well as exerting immunomodulator effects, notably stimulating the production of cytokines and the activation of T cells and natural killer cells [10]. Lenalidomide also has anti-angiogenic properties and is a particularly attractive option for maintenance treatment of MM. Indeed, a number of comprehensive review studies have reported positive findings regarding the use of lenalidomide in the treatment (both initial and maintenance) of MM in recent years [10], [15].

To gain a better, more complete understanding of the efficacy and safety of lenalidomide, we performed a meta-analysis of randomized controlled trials in which patients with MM received lenalidomide as initial or maintenance therapy.

## Materials and Methods

### Search Strategy

PubMed, EMBASE, CANCERLIT, SCOPUS, and the Cochrane central register of controlled trials were searched using the terms “lenalidomide or revlimid AND multiple myeloma.”The ‘related articles’ function in PubMed was used to identify other potentially relevant articles. Further, we attempted to identify other potentially relevant articles by searching the reference sections of pertinent manuscripts and by contacting known experts in the field. We also searched the ClinicalTrials.gov registry (http://clinicaltrials.gov/). No language restrictions were applied. The last search was performed in November 2012.

### Selection Criteria

To be included in the analyses, studies were required to be randomized controlled trials that evaluated initial or maintenance therapeutic outcomes of lenalidomide for the treatment of MM. Studies were also required to report the criteria used for selecting patients, the treatment strategy, and the definition and evaluation of therapeutic outcomes. Studies were excluded from our analyses if the outcomes of interest were not clearly reported or if duplicate reporting of patient cohorts was apparent.

### Data Extraction and Methodological Quality Appraisal

Two independent reviewers extracted trial details pertaining to the participants, inclusion and exclusion criteria, the lenalidomide treatment protocol, prognostic outcomes, and adverse events. The information extracted by the two reviewers was compared and any disagreements were resolved by consultation with a third reviewer.

The quality of studies was assessed using the “risk of bias” method recommended by the Cochrane Collaboration [16]. In addition, two reviewers independently appraised the methodological quality of each trial by examining the adequacy of the randomization, allocation concealment, blinding, number of drop-outs, other risks of bias, and whether intention-to-treat analysis had been carried out.

### Outcomes Assessments

The efficacy of lenalidomide treatment was evaluated according to the criteria of the European Group for Blood and Marrow Transplantation [17] or the International Uniform Response Criteria for MM [18]. A partial response was defined as a reduction of M protein by at least 50% in serum, 90% in urine, or both. A complete response was defined as the complete disappearance of M protein in serum and urine on immunofixation if confirmed by bone marrow evaluation. A very good partial response was defined as the complete disappearance of M protein in serum and urine on immunofixation in the absence of bone marrow evaluation.

Progression-free survival was measured from randomization to the date of the first assessment showing disease progression. Overall survival was calculated as the time from randomization until death from any cause. Safety outcomes included the incidence of adverse events, specifically neutropenia, anemia, thrombocytopenia, deep vein thrombosis, neuropathy, infection, and second primary cancer.

### Statistical Analysis

Analyses were conducted using the Review Manager version 5.1 (Cochrane Collaboration, Oxford, England) and were performed according to PRISMA guidelines [19]. The effect of treatment for each study is expressed as a hazard ratio (HR) of the lenalidomide treatment arm over the non-lenalidomide treatment arm. Effect sizes of dichotomous outcomes are reported as risks ratios (RR) with 95% confidence intervals. A pooled estimate of the HR and RR was determined using the DerSimonian and Laird random-effect model [20]. Data were only pooled for trials that exhibited adequate clinical and methodological similarity. Statistical heterogeneity was assessed using the I^2^ test, with I^2^quantifying the proportion of the total outcome variability attributable to variability among the trials. Statistical significance was indicated by *P*<0.05.

## Results

### Selection of the trials

**Figure 1:**
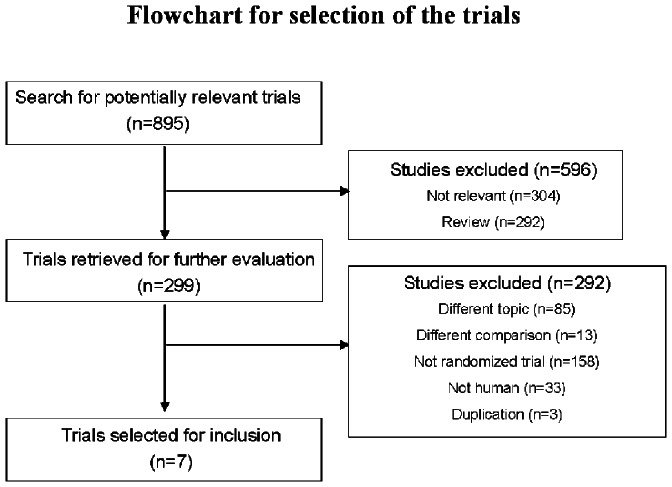
Flowchart of the selection of studies.

Our initial search yielded 895 potentially relevant trials, of which 596 were deemed ineligible after title and abstract screening (Figure 1). The full text of 299 trial reports were reviewed in full. Most of these were subsequently excluded (n = 292), leaving a total of seven trials that were included in the meta-analysis [21]–[27].

### Characteristics of the trials

The characteristics of the trials included in the meta-analysis are summarized in Table 1. The trial results were published between 2007 and 2012 and had sample sizes ranging from 192 to 614 participants. The two treatment groups were relatively similar in terms of participants ages across all seven trials. Four of the trials investigated whether the addition of lenalidomide therapy improved outcomes for participants with MM who had received at least one previous antimyeloma therapy [24]–[27]. Two trials investigated the initial therapeutic effect of lenalidomide in participants with newly diagnosed MM [23] or those ineligible for transplantation [21]. One study investigated if the efficacy of lenalidomide plus high-dose dexamethasone could be preserved, but corresponding toxicity reduced, with a lower dexamethasone dose in participants with untreated symptomatic MM [22]. The protocol for lenalidomide treatment differed between the studies. Notably, control group participants in the trial reported by Zonder et al. [23] were encouraged to cross over to the open-label lenalidomide treatment group upon disease progression.

**Table 1:** Characteristics of studies fulfilling inclusion criteria in the meta-analysis.

Author [Year]	Inclusion criteria	No. of patients (% of male)	Age, mean (range)		Intervention
**Initial treatment**					
Palumbo [2012]	Patients with MM ineligible for transplantation	MPR-R: 152/MPR: 153/MP: 154		MPR-R: 71 (65–87)/MPR: 71 (65–86)/MP: 72 (65–91)	MPR-R: L maintenance, 10 mg on day1-21 of each 28-d cycle/MPR: P maintenance/MP: P during induction and maintenance
Rajkumar [2010]	Untreated symptomatic MM	L+ high D: 223 /L+ low D: 222		L+ high D: 66 (36–87)/L+ low D: 65 (35–85)	L+ high D: L 25 mg on day 1–21+ D 40 mg on d 1–4, 9–12, and 17–20 of a 28-d cycle/L+ low D: L 25 mg on day 1–21+ D 40 mg on d 1, 8, 15, and 22of a 28-d cycle
Zonder [2010]	Newly diagnosed MM	L: 97 (55)/P: 95 (58)		Age >/65y.o L: 49%/P: 47%	35-day induction cycle with D 40 mg/d on day 1–4, 9–12, and 17–20+ L 25 mg/d for 28 days. Maintenance with D 40 mg/d on day 1–4 and 15–18+ L 25 mg/d for 21 days
**Second-line therapy**					
Attal [2012]	Nonprogressive MM after first-line transplantation	L: 307 (55)/P: 307 (59)	L: 55 (22–67) /P: 55(32–66)		Consolidation therapy with L 25 mg/d, on day 1–21 of each 28-day cycle x 2 cycles, followed by L 10 mg/d for the first 3 months, increased to 15mg if tolerated
Dimopoulos [2007]	Relapsed or refractory MM, at least one previous antimyeloma therapy	L: 176 (59.1)/P: 175 (58.9)	L: 63 (33–84) /P: 64(40–82)		L 25 mg, on day 1 to 21 of a 28-day cycle + D 40 mg/d on day 1–4, 9–12, and 17–20 for the first 4 cycles, after the 4^th^ cycle, only on day 1–4
McCarthy [2012]	Patients with MM after stem-cell transplantation	L: 231 (52.4)/P: 229 (56.3)	L: 59 (29–71)/P: 58 (40–71)		L 10 mg/d, 100 days after stem-cell transplantation
Weber [2007]	Patients who had received at least one previous therapy for MM	L: 177 (59.9)/P: 176 (59.1)	L: 64 (36–86) /P: 62(37–85)		L 25 mg on day 1–21 of a 28-d cycle + D 40 mg/d on day 1–4, 9–12, and 17–20 for the first 4 cycles, after the 4^th^ cycle, only on day 1–4

D: dexamethasone; L: lenalidomide; MM: multiple myeloma; P: placebo; MPR (melphalan-prednisone-lenalidomide): nine 28-d cycles of melphalan (at a dose of 0.18 mg/kg of body weight on day 1–4), prednisone (2 mg/kg on day 1–4), and lenalidomide (10 mg on days 1–21).

The methodological quality of the trials included in the meta-analysis is summarized in Table 2. Two trials reported acceptable methods of randomization [22], [24]. Only one trial described the method of allocation concealment [20]. Five trials reported blinding of the participants and outcome assessors [21], [24]–[27]. Six trials used an intention-to-treat analysis [21]–[27]. The number of participant drop-outs was acceptable (<20%) in the majority of the trials. Other biases that existed in the trials included: early stopping of lenalidomide maintenance therapy based on an increased incidence of adverse events [23], [25]; early trial unblinding and crossover [23], [24], [26], [27]; trial designed and data analyzed by the manufacturer of lenalidomide [21], [26]; and patients receiving inappropriate doses of steroid treatment [22].

**Table 2:** Methodological quality assessment of included trial.

Author [Year]	Location	Allocation generation	Allocation concealment	Double blinding	Data analysis	Drop-out	Other risk of bias
**Initial treatment**							
Palumbo [2012]	Europe, Israel, Australia	Unclear	Unclear	Double blinded	PP	38.1% not entered maintenance phase	Study designed and data analysis by manufacturer
Rajkumar [2010]	United States	Computer generated	Adequate	Open-label	ITT	5.2%	Patients received inappropriately high-dose steroids beyond the first four cycles
Zonder [2010]	United States	Unclear	Unclear	Open-label	PP/ITT	1.0% not entered in adverse event evaluation	Patients in control group could cross-over to lenalidomide group on disease progression; early study closure
**Second-line therapy**							
Attal [2012]	France, Belgium, Switzerland	Unclear	Unclear	Double blinded	ITT	7.0%	Early stopping lenalidomide maintenance therapy based on an increased incidence of second primary cancers
Dimopoulos [2007]	Europe, Israel, Australia	Unclear	Unclear	Double blinded	ITT	N/A	Study designed and data analysis by manufacturer, early study data are unblended
McCarthy [2012]	United States	Unclear	Unclear	Double blinded	ITT	N/A	Increase in time to progression led to early study unblinding and crossover
Weber [2007]	United States, Canada	Computer generated	Unclear	Double blinded	ITT	N/A	Response rate and time to progression are based on data obtained before unblinding

ITT, intention-to-treat; PP, per-protocol; N/A, not available.

### Response rate

**Figure 2:**
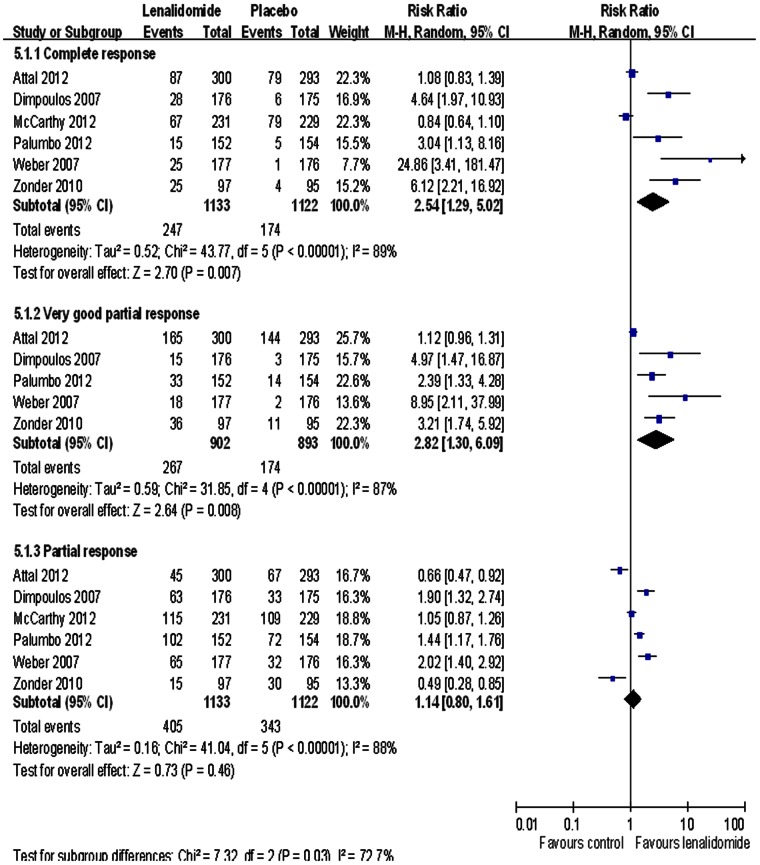
Individual trials and overall risk ratios for response rates (complete response, very good partial response, and partial response) in the comparison of lenalidomide and placebo. Squares on the risk ratio plot are proportional to the weight of each study, which is based on the Mantel-Haenszel (M-H) method. Risk ratios are presented with 95% confidence intervals (CIs).

All trials reported response rate outcomes with and without lenalidomide treatment. We included data from six of the trials in our analysis and excluded the data from one trial that did not compare lenalidomide and placebo groups [22]. We extracted data from the melphalan – prednisone – lenalidomide induction followed by lenalidomide maintenance (MPR-R) group and the melphalan – prednisone followed by placebo (MPR)group for pooling in the trial reported by Palumbo et al. [21]. Overall, we found **a** significant difference between the two treatment groups, with more patients in the lenalidomide group experiencing greater complete response (RR = 2.54; 95% CI: 1.29 to 5.02), and very good partial response (RR = 2.82; 95% CI: 1.32 to 6.09) (Figure 2). There were no significant effects of treatment in the partial response RR. There was significant heterogeneity among the trials for complete response (I^2^ = 89%), very good partial response (I^2^ = 87%), and partial response (I^2^ = 88%).

### Progression-free survival

As first-line treatment for patients with newly diagnosed MM, Palumbo et al. [21] reported that MPR-R was associated with significantly increased progression-free survival (31 months) compared with MPR (14 months; HR: 0.49; *P*<0.001) or melphalan – prednisone (13 months; HR: 0.40; *P*<0.001)]. Zonder et al. [23] also confirmed the superiority of lenalidomide plus dexamethasone over placebo plus dexamethasone as a first-line therapy for MM as indicated by an increased rate of one-year progression-free survival (78% vs 52%, *P* = 0.002).

**Figure 3:**
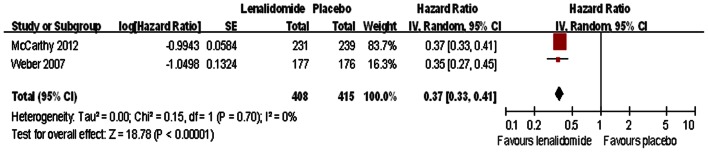
Individual trials and overall hazard ratios for progression-free survival in the comparison of lenalidomide and placebo. Squares on the hazard ratio plot are proportional to the weight of each study, which is based on the inverse variance (IV) method. Hazard ratios are presented with 95% confidence intervals (CIs).

As second-line treatment, three trials compared progression-free survival in participants treated with lenalidomide and placebo [24], [25], [27]. We used data obtained from two of these trials in our meta-analysis [24], [27], and excluded the data from one of the trials because of inadequate data for pooling [25]. A random-effects statistical model revealed thatlenalidomide therapy was associated with increased progression-free survival compared with placebo (HR  = 0.37; 95% CI: 0.33–0.41) (Figure 3). There was no evidence of significant heterogeneity among the trials (I^2^ = 0%). Attal et al. [25] reported that lenalidomide maintenance therapy improved median progression-free survival (41 vs 23 months with placebo, *P*<0.001). In the trial reported by Dimopoulos et al. [26], the time to progression was significantly increased in the group of patients who received lenalidomide plus dexamethasone (11.3 vs 4.7 months with placebo, *P*<0.001).

### Overall survival

Palumbo et al. [21] reported that the effect of continuous lenalidomide treatment on overall survival in participants with newly diagnosed MM was unclear. Zonder et al. [23] reported that the one-year overall survival rate was similar in a comparison of participants who were treated with lenalidomide plus dexamethasone or placebo plus dexamethasone.

**Figure 4:**
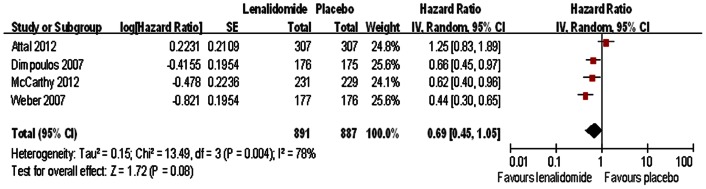
Individual trials and overall hazard ratios for overall survival in the comparison of lenalidomide and placebo. Squares on the hazard ratio plot are proportional to the weight of each study, which is based on the inverse variance (IV) method. Hazard ratios are presented with 95% confidence intervals (CIs).

As second-line treatment, four trials compared the rate of overall survival in participants treated with lenalidomide and placebo [24]–[27]. Although there was a trend for increased overall survival with lenalidomide, our meta-analysis revealed that there was no statistically significant difference in overall survival between lenalidomide maintenance therapy and placebo (HR  = 0.69; 95% CI: 0.41–1.05) (Figure 4). There was evidence of significant heterogeneity among the trials (I^2^ = 78%).

### Adverse outcomes

**Figure 5:**
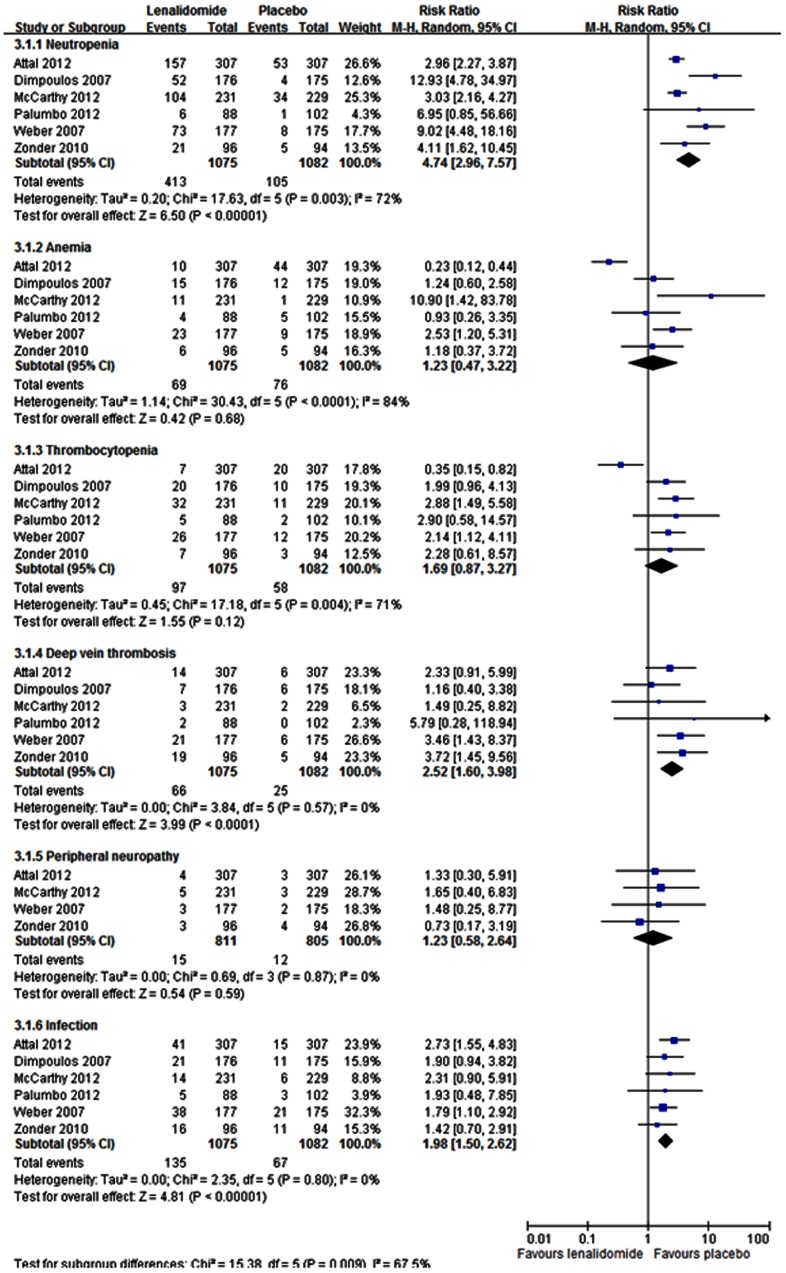
Individual trials and overall risk ratios for the incidence of adverse events (neutropenia, anemia, thrombocytopenia, deep vein thrombosis, peripheral neuropathy, and infection) in the comparison of lenalidomide and placebo. Squares on the risk ratio plot are proportional to the weight of each study, which is based on the Mantel-Haenszel (M-H) method. Risk ratios are presented with 95% confidence intervals (CIs).

All trials reported the incidence of adverse events. Data from six of the trials were included in our analysis; the data from one trial that had not compared lenalidomide and placebo groups were excluded [22]. Overall, we found significant differences between the two treatment groups, with more patients in the lenalidomide group experiencing greater incidence of neutropenia (RR = 4.74; 95% CI: 2.96 to 7.57), deep vein thrombosis (RR = 2.52; 95% CI: 1.60 to 3.98), and infection (RR = 1.98; 95% CI: 1.50 to 2.62) (Figure 5). No significant effects of lenalidomide were noted on the RR of anemia, thrombocytopenia, and peripheral neuropathy.

### Second primary cancers

**Figure 6:**
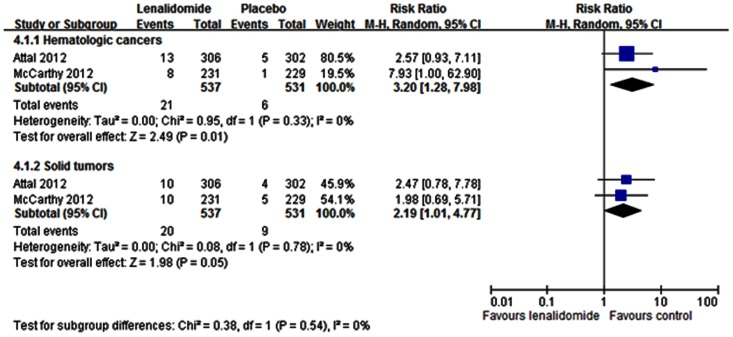
Individual trials and overall risk ratios for the incidence of second primary cancers in the comparison of lenalidomide and placebo. Squares on the risk ratio plot are proportional to the weight of each study, which is based on the Mantel-Haenszel (M-H) method. Risk ratios are presented with 95% confidence intervals (CIs).

Two trials reported the incidence of second primary cancers [25], [27]. Overall, lenalidomide increased the RR for hematologic cancers (*P* = 0.01, Figure 6). We found a significant difference between the two treatment groups, with more patients in the lenalidomide group experiencing greater incidence of new hematologic cancers (RR = 3.20; 95% CI: 1.28 to 7.98), and solid tumors (RR = 2.19; 95% CI: 1.01 to 4.77) (Figure 6). No evidence of significant heterogeneity was noted among the trials for hematologic cancer (I^2^ = 0%) or solid tumors (I^2^ = 0%).

## Discussion

During the last five years, a number of RCTs have examined the efficacy and safety of lenalidomide for the treatment of MM. Hence, we performed a meta-analysis in an attempt to gain further insight into the efficacy and safety of this treatment. A total of seven RCTs met the criteria for inclusion in our meta-analysis. The included trials were heterogenous in terms of inclusion criteria and treatment regimens; however, our overall analyses revealed that lenalidomide therapy significantly improved the rates of complete response and partial response and, importantly, increased progression-free survival relative to placebo/control. These findings were consistent among all RCTs included in our study [21]–[27]. In contrast, lenalidomide significantly increased the risk of several adverse events, specifically neutropenia, deep vein thrombosis, infection, and hematologic cancer.

More recent studies report conflicting results. Gay et al. [28] retrospectively studied 411 patients to compare the efficacy and toxicity of lenalidomide plus dexamethasone versus thalidomide plus dexamethasone as initial therapy for newly diagnosed myeloma. In that study report, patients receiving lenalidomide plus dexamethasone had a longer time to progression, progression-free survival, and overall survival than the group receiving thalidomide plus dexamethasone. A recent observational study assessed the efficacy and safety of lenalidomide plus dexamethasone in patients with relapsed or refractory MM who had been previously treated with thalidomide; the group receiving lenalidomide plus dexamethasone experienced a higher overall response rate, longer time to progression, and progression-free survival compared to those receiving placebo plus dexamethasone, despite prior thalidomide exposure [29]. Clearly, further RCTs are needed to determine if specific lenalidomide treatment regimens and/or patients characteristics are more likely to result in significantly increased overall survival.

In addition to efficacy, safety is an equally important consideration for any chemotherapeutic agent. Obviously, the balance of any treatment must favor benefit over harm. The majority of adverse events reported in the studies we evaluated (i.e., neutropenia, deep vein thrombosis, infection, and hematologic cancer) are manageable and do not appear to outweigh the benefits of treatment. Neutropenia and other hematologic toxicities can be managed with dose adjustment and/or treatment with granulocyte colony stimulating factor [21], [25]–[26], [30]. Thromboprophylaxis is clearly indicated for patients being treated with lenalidomide to ameliorate the risk of deep vein thrombosis and other thrombolytic events [24], [26], [30]. The optimal prophylactic agent is yet to be identified [30] and inevitably must be determined on a case-by-case basis; however, a recent study found acetylsalicylic acid was an effective thromboprophylactic in patients treated with lenalidomide who had a low thromboembolic risk [31]. The increased risk of infection with lenalidomide treatment suggests that antibiotic prophylaxis should be considered as part of the treatment regimen [24], [30]. Increased risk of hematologic cancer with lenalidomide treatment is a concern, but is not unexpected [21], and highlights the importance of close monitoring for early detection of second cancers. Regarding the significant heterogeneity between the included studies, First, it must be noted that the dosage, duration and program of lenalidomide treatment differed across the studies. Second, the characteristics of individual patients in terms of MM severity could potentially affect the evaluated outcomes. Third, the primary induction therapy for MM differed greatly among the studies we reviewed. Additionally, variability in clinical factors and non-uniform reporting of clinical parameters contributed to measurement bias. This variability clearly emphasizes the need for further research to determine optimal lenalidomide doses and therapeutic regimens individualized according to patients' characteristics.

The strengths of our review include the comprehensive search for eligible studies, the systemic and explicit application of eligibility criteria, the careful consideration of study quality, and the rigorous analytical approach. However, our review is limited by the methodological quality of the original studies (Table 2). First, only two of the included studies reported an adequate technique for randomized allocation [22], [24]. Second, early discontinuance of lenalidomide maintenance therapy based on an increased incidence of adverse events may influence the statistical power of therapeutic outcomes [23], [25]. Finally, population characteristics, crossover designs with the probable risk of inadequate washout period, differing lenalidomide schedules and dosages, and use of concomitant drugs may have resulted in a somewhat speculative interpretation of our analysis. Also, patients' ages in the included studies ranged from 22 to 91 years, and efficacy in older individuals is not necessarily the same as in younger individuals. Separate subgroup analysis should be done for older vs. younger adults, but the data needed to conduct subgroup analysis could not be extracted from the studies. Further, because the seven trials we reviewed compared lenalidomide therapy with placebo, and not with thalidomide, no conclusion can be made regarding lenalidomide as first-line treatment over thalidomide.

In summary, the findings from our meta-analysis indicate that lenalidomide therapy significantly improves response rates and increases progression-free survival in patients with newly diagnosed MM, and those receiving previous antimyleoma therapy, but it is associated with an increased risk of a number of adverse events. Obviously, pros and cons remain on the clinical efficacy of lenalidomide as first-line treatment for MM. Essentially, while lenalidomide is an effective treatment for MM, the likely associated adverse events must be considered for each case and appropriate dose adjustments and/or prophylactic treatment initiated where possible. Further research is needed to determine optimal lenalidomide treatment regimens and combinations and the patients most likely to benefit.
